# SET8 is a novel negative regulator of TGF-β signaling in a methylation-independent manner

**DOI:** 10.1038/s41598-023-49961-x

**Published:** 2023-12-18

**Authors:** Mai Nagasaka, Yasumichi Inoue, Yuji Nagao, Chiharu Miyajima, Daisuke Morishita, Hiromasa Aoki, Mineyoshi Aoyama, Takeshi Imamura, Hidetoshi Hayashi

**Affiliations:** 1 Department of Cell Signaling, Graduate School of Pharmaceutical Sciences, Nagoya City University, Nagoya, 467-8603 Japan; 2 Department of Pathobiology, Graduate School of Pharmaceutical Sciences, Nagoya City University, Nagoya, 467-8603 Japan; 3 Department of Molecular Medicine for Pathogenesis, Graduate School of Medicine, Ehime University, Ehime, 791-0295 Japan

**Keywords:** Cancer, Oncogenes

## Abstract

Transforming growth factor β (TGF-β) is a multifunctional cytokine that induces a diverse set of cellular processes principally through Smad-dependent transcription. Transcriptional responses induced by Smads are tightly regulated by Smad cofactors and histone modifications; however, the underlying mechanisms have not yet been elucidated in detail. We herein report lysine methyltransferase SET8 as a negative regulator of TGF-β signaling. SET8 physically associates with Smad2/3 and negatively affects transcriptional activation by TGF-β in a catalytic activity-independent manner. The depletion of SET8 results in an increase in TGF-β-induced plasminogen activator inhibitor-1 (PAI-1) and p21 expression and enhances the antiproliferative effects of TGF-β. Mechanistically, SET8 occupies the *PAI-1* and *p21* promoters, and a treatment with TGF-β triggers the replacement of the suppressive binding of SET8 with p300 on these promoters, possibly to promote gene transcription. Collectively, the present results reveal a novel role for SET8 in the negative regulation of TGF-β signaling.

## Introduction

Transforming growth factor-β (TGF-β) is a multifunctional cytokine that regulates a diverse set of cellular processes, including cell proliferation, migration, differentiation, and apoptosis^1–3^. Aberrant TGF-β signaling has been implicated in the pathogenesis of various diseases, including cancer^4–6^. In most epithelial cell types, TGF-β functions as a tumor suppressor by inducing cell cycle arrest, senescence, and apoptosis. A number of human cancers reportedly have mutations that disable a component of the TGF-β signaling network, such as inactive mutations in *TGFBR2* in colorectal cancer and *Smad4* in pancreatic carcinomas^7,8^. However, during tumor progression, overtly malignant cells may evade the tumor-suppressive effects of TGF-β and exploit it for their advantage, such as metastasis. Since impairments in these effects of TGF-β may contribute to cancer progression, further studies are needed to elucidate the regulatory mechanisms underlying pleiotropic TGF-β signaling.

A TGF-β ligand initiates signaling by binding to two pairs of receptor serine/threonine kinases, known as TGF-β type I receptor (TβRI) and type II receptor (TβRII), and triggers receptor activation and the phosphorylation of Smad transcription factors^9,10^. The canonical TGF-β signaling pathway uses receptor-activated Smad2 and Smad3, which form heterotrimeric complexes with Smad4^11,12^. This complex translocates into the nucleus and regulates TGF-β target gene expression by cooperating with other transcriptional coactivators or corepressors. Due to the low affinity of the activated Smad complex binding to DNA, it must interact with other transcription factors to form stable complexes with DNA and elicit specific transcriptional regulation^13,14^. In addition, histone-modifying enzymes have been reported to facilitate assessments of the specificity of Smad-dependent transcription by changing chromatin accessibility patterns^15^.

SET domain-containing protein 8 (also known as KMT5A, Pr-Set7/9, and SETD8), a member of the SET domain-containing methyltransferase family, is the sole enzyme targeting histone H4 lysine 20 monomethylation (H4K20me1)^16,17^. In addition to histone modifications, SET8 has been shown to monomethylate non-histone proteins, including p53, Numb, and PCNA^18–20^. SET8 has been implicated in vital cellular processes, such as DNA replication, mitosis, DNA damage repair, and gene regulation^21^. SET8 protein expression is tightly regulated during the cell cycle, and *SET8* homozygous null mutant embryos displayed early lethality due to cell-cycle defects and improper mitotic chromosome condensation, reflecting its essential roles in the proper progression of the cell cycle^22^. The dysregulation of SET8 expression has been implicated in many disease conditions, including cancer^22,23^. SET8 overexpression has been detected in various types of tumors and associated with the poor survival rate of cancer patients^24–26^. SET8 has been suggested to play a role in cancer proliferation, migration, and oncogenesis.

We herein identified SET8 as a novel negative regulator of TGF-β signaling. SET8 interacted with Smad2/3 and negatively affected transcriptional activation by TGF-β in a catalytic activity-independent manner. The depletion of endogenous SET8 increased TGF-β-induced plasminogen activator inhibitor-1 (PAI-1) and p21 expression and augmented the antiproliferative effects of TGF-β. Mechanistically, SET8 occupied the *PAI-1* and *p21* promoters, and a treatment with TGF-β triggered the replacement of the suppressive binding of SET8 with p300 on these promoters to facilitate gene transcription. The present results suggest that SET8 has an essential function in the suppression of TGF-β-mediated cytostasis, and also that the SET8 status may be involved in the regulatory modes of TGF-β signaling.

## Results

### SET8 physically associates with Smad2 or Smad3

**Figure 1:**
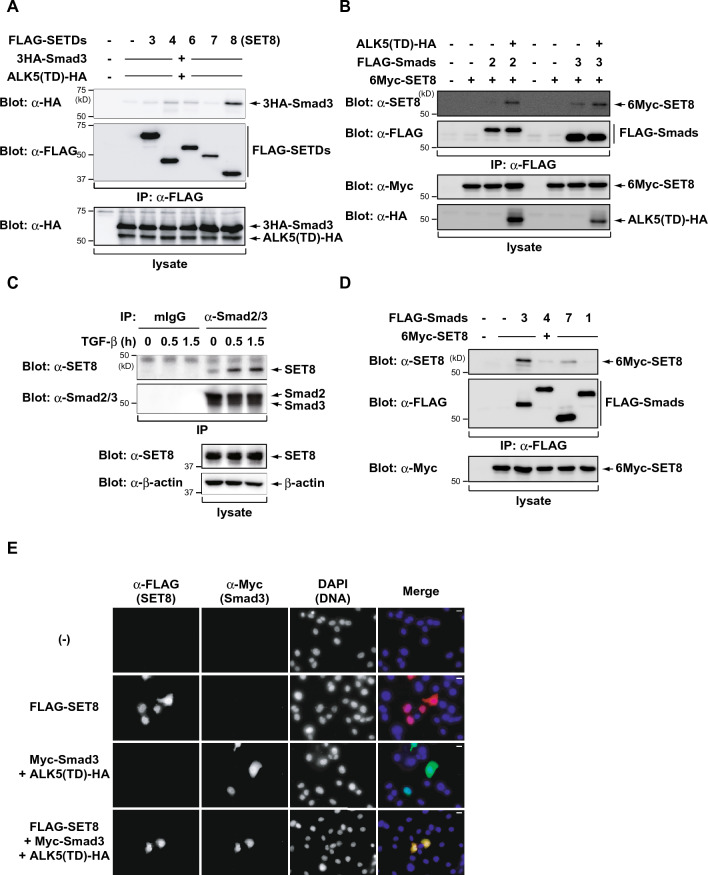
SET8 physically associates with Smad2 or Smad3. (**A**,**B**) COS7 cells were transiently transfected with the indicated constructs. After 24 h, cell lysates were immunoprecipitated (IP) with an anti-FLAG antibody and then immunoblotted with the indicated antibodies. (**C**) HepG2 cells were treated with or without 100 pM of TGF-β for the indicated periods. Cell lysates were immunoprecipitated (IP) with an anti-Smad2/3 antibody and then immunoblotted with the indicated antibodies. (**D**) COS7 cells were transiently transfected with 6Myc-SET8 in the presence of FLAG-Smads. After 24 h, cell lysates were immunoprecipitated (IP) with an anti-FLAG antibody and then immunoblotted with the indicated antibodies. (**E**) COS7 cells were transiently transfected with indicated plasmids and stained with an anti-FLAG antibody for SET8 (Alexa 594, red) and anti-Myc antibody for Smad3 (FITC, green). Scale bars; 10 μm.

In an effort to elucidate the regulatory mechanisms of TGF-β signaling, we initially examined the SET domain-containing histone lysine methyltransferases, such as SET domain-containing protein (SETD) and SET and MYND domain-containing protein (SMYD), which play a pivotal role in the epigenetic regulation of gene expression^27^. To identify Smad3-interacting proteins, COS7 cells were transfected with 3HA-Smad3, several FLAG-SETDs or FLAG-SMYDs, and ALK5 (T204D)-HA, a constitutively active TβRI mutant^28^, and the immunoprecipitation assay was performed with the anti-FLAG antibody. As shown in Figs. 1A and S1, SET8 significantly associated with Smad3. Ectopically expressed SET8 also associated with Smad2. In contrast, the interaction between SET8 and Smad2 or Smad3 was minimal in untreated cells, but was markedly enhanced by the co-expression of ALK5 (T204D)-HA (Fig. 1B). Furthermore, we detected an interaction between endogenous SET8 and Smad2/3 in response to TGF-β in HepG2 cells (Fig. 1C). In HepG2 cells, binding of Smad2/3 to SET8 was enhanced at 30 min after TGF-β treatment, and was maintained at 1.5 h. SET8 binding to other Smad family members was not significant, indicating that SET8 specifically associated with Smad2 or Smad3 (Fig. 1D). To further confirm the interaction between SET8 and Smad3, we investigated the subcellular localization of SET8 and Smad3. In Fig. 1E, FLAG-SET8 expressed in COS7 cells was mainly localized in the nucleus and partially expressed in the cytoplasm. On the other hand, Myc-Smad3 localized to both the cytoplasm and nucleus when co-expressed with the ALK5 (T204D)-HA. Co-expression of Myc-Smad3 and FLAG-SET8 with the ALK5 (T204D)-HA resulted in co-localization mostly in the nucleus and some in the cytoplasm (Fig. 1E). These results showed that SET8 physically associated with Smad2 or Smad3 in cells.

### The N-terminal domain of SET8 binds to the MH2 domain of Smad3

**Figure 2:**
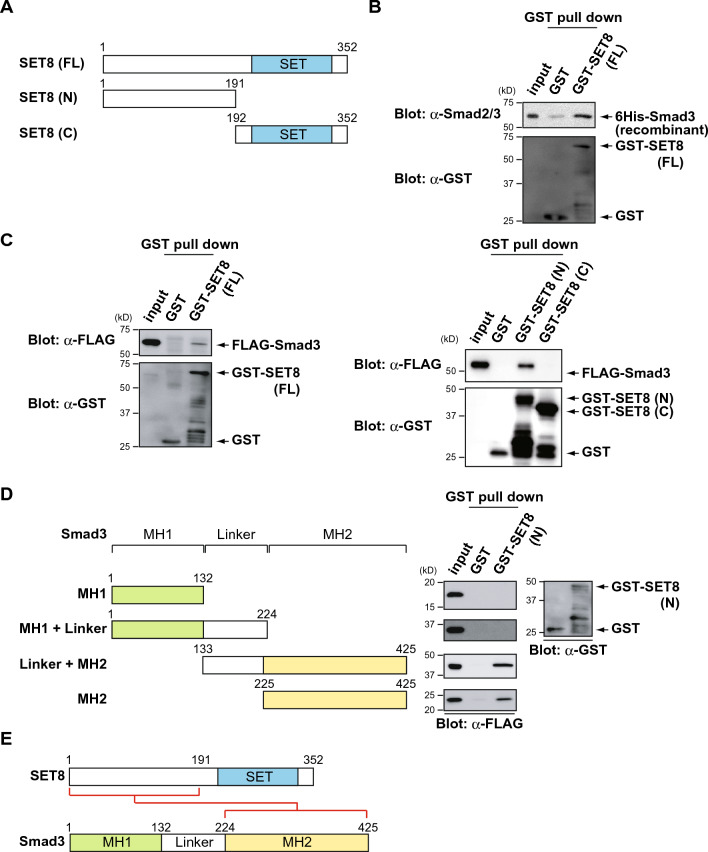
The N-terminal domain of SET8 binds to the MH2 domain of Smad3. (**A**) Schematic representation of full-length and deletion mutants of SET8. (**B**) In vitro binding assay for recombinant 6His-Smad3 and GST-SET8. (**C**) HEK293 cells were transfected with FLAG-Smad3. After 48 h, cell lysates were incubated with GST or GST-SET8 (FL, N, and C) and subjected to a GST pull-down assay, followed by immunoblotting with an anti-FLAG antibody. (**D**) HEK293 cells were transfected with FLAG-Smad3 deletion mutants. After 48 h, cell lysates were incubated with GST or GST-SET8 (N) and subjected to a GST pull-down assay, followed by immunoblotting with an anti-FLAG antibody. (**E**) Schematic representation of protein–protein interactions between SET8 and Smad3. The interacting domains are connected with a red line.

We investigated the interaction between SET8 and Smad3 in more detail using full-length and deletion mutants of SET8 (Fig. 2A). The results of an in vitro GST pull-down assay showed that recombinant GST-SET8 and recombinant 6His-Smad3 bound to each other, suggesting a direct interaction (Fig. 2B). In an effort to map the interaction interface of SET8 with Smad3, an in vitro GST pull-down assay was performed with cell extracts containing FLAG-Smad3 expressed in COS7 cells and GST-SET8 (FL), the GST-SET8 N-terminal region (1–191 amino acids (a.a.)), and GST-SET8 C-terminal SET domain (192–352 a.a.). SET8 has the catalytic SET domain in its C terminus, the only recognizable motif in this protein^17^. SET8 reportedly binds to various proteins, including PCNA and TWIST, via its N terminus^20,29^. As expected, the N-terminal region of SET8 was essential for binding to Smad3 (Fig. 2C). We also performed an in vitro GST pull-down assay using various deletion mutants of Smad3. Smad3 contained two conserved structural domains: the N-terminal Mad Homology 1 (MH1) domain and the C-terminal MH2 domain connected by a linker region^2^. The MH1 and MH2 domains both reportedly interacted with various proteins in the nucleus^1^. As shown in Fig. 2D, the MH2 domain of Smad3 was specifically pulled down with GST-SET8 (N). Collectively, these results suggest that the N-terminal region of SET8 interacted with the MH2 domain of Smad3 (Fig. 2E).

### SET8 negatively affects transcriptional activation by TGF-β

**Figure 3:**
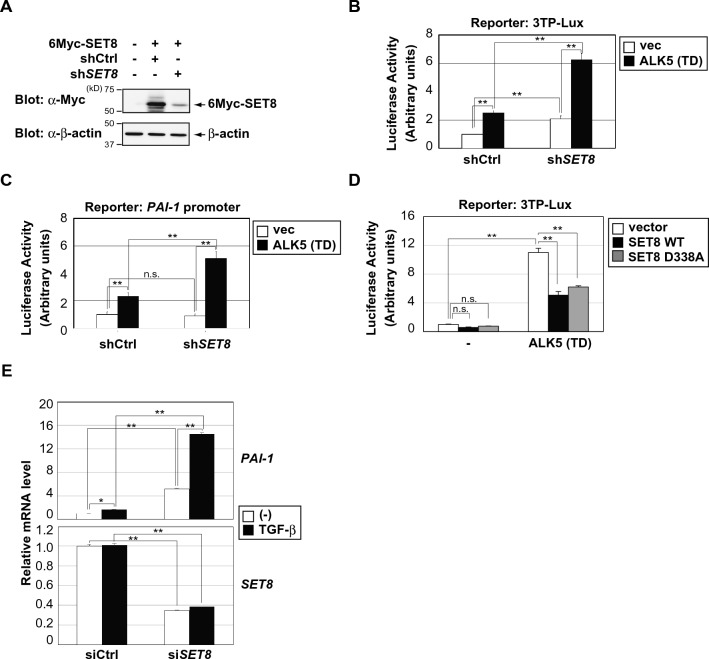
SET8 negatively affects transcriptional activation by TGF-β. (**A**) HEK293 cells were transfected with the indicated constructs and shRNAs. After 48 h, cell lysates were analyzed by immunoblotting with the indicated antibodies. (**B**,**C**) Effects of the SET8 knockdown by shRNA on the activation of 3TP-Lux (**B**) and the *PAI-1* promoter (**C**) induced by ALK5 (T204D) in HEK293 cells. Results are shown as means ± S.D. (n = 3). (**D**) Effects of the catalytically inactive mutant of SET8 (D338A) on the activation of 3TP-Lux induced by ALK5 (T204D) in H1299 cells. Results are shown as means ± S.D. (n = 3). (**E**) HepG2 were transiently transfected with the indicated siRNAs. After 48 h, cells were treated with 40 pM of TGF-β for 6 h. The expression of each gene was assessed by quantitative PCR, and the mRNA levels of the indicated genes were normalized with *HPRT1* mRNA. Results are shown as means ± S.D. (n = 3). Significant differences are indicated as **p < 0.01, *p < 0.05, n.s.: not significant.

To examine whether SET8 affects Smad-dependent signaling mediated by TGF-β, we knocked down SET8 expression in HEK293 cells (Fig. 3A) and performed a luciferase assay based on TGF-β-responsive reporters. The reporter 3TP-Lux contains the − 740/− 636 region of the *PAI-1* promoter and three repeats of the TPA-responsive element and is often used to test the transcriptional activity of TGF-β/Smad signaling^30^. The expression of ALK5 (T204D) enhanced 3TP-Lux activity, which was also augmented by the knockdown of SET8 (Fig. 3B). Similarly, the knockdown of SET8 markedly increased *PAI-1* promoter activity induced by ALK5 (T204D) (Fig. 3C). On the other hand, the overexpression of SET8 decreased the reporter activity of 3TP-Lux induced by ALK5 (T204D). We also found that a catalytically inactive SET8 (D338A) mutant repressed the ALK5 (T204D)-induced promoter activity of 3TP-Lux as effectively as wild-type SET8, implying that SET8 exerts effects on TGF-β target promoters that are independent of its catalytic activity (Fig. 3D). Moreover, the knockdown of SET8 enhanced the TGF-β-induced transcription of *PAI-1* more than control siRNA in HepG2 cells (Fig. 3E). Of note, TGF-β induced similar levels of Smad2 and Smad3 phosphorylation in both control and SET8 knockdown HepG2 cells, suggesting that the molecular mechanism of action of SET8 resides after the phosphorylation events of Smad2 and Smad3 (Fig. S2). These results indicate that SET8 negatively regulated the transcriptional activation of TGF-β.

### SET8 down-regulates the TGF-β-induced expression of PAI-1 and p21 and represses antiproliferative effects of TGF-β

**Figure 4:**
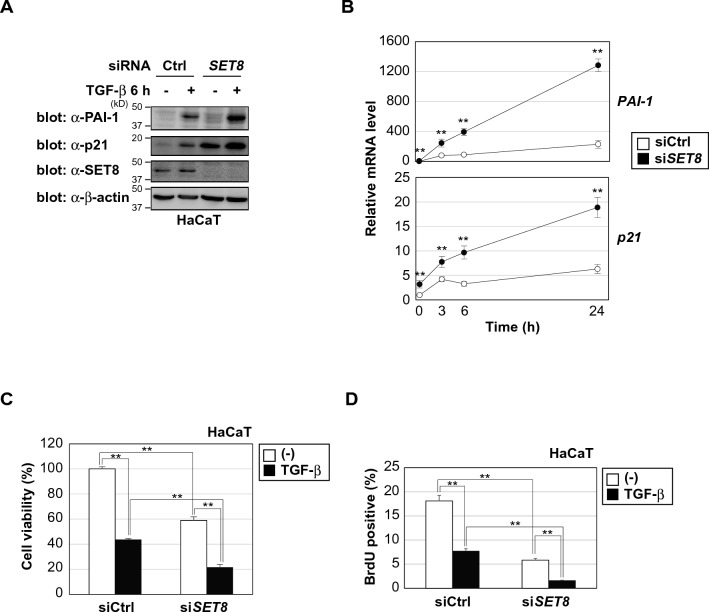
SET8 down-regulates the TGF-β-induced expression of PAI-1 and p21 and represses antiproliferative effects of TGF-β. (**A**) HaCaT cells were transiently transfected with the indicated siRNAs. After 48 h, cells were treated with 40 pM of TGF-β for 6 h. Cell lysates were immunoblotted with the indicated antibodies. (**B**) HaCaT cells were transiently transfected with the indicated siRNAs. After 48 h, cells were treated with 40 pM of TGF-β for the indicated times. The expression of each gene was assessed by quantitative PCR, and the mRNA levels of the indicated genes were normalized with *HPRT1* mRNA. Results are shown as means ± S.D. (n = 3). (**C**) HaCaT cells were transiently transfected with the indicated siRNAs and treated with 100 pM of TGF-β. After 72 h, cell viability was measured by a WST-8 cell proliferation assay. Results are shown as means ± S.D. (n = 3). (**D**) HaCaT cells were transiently transfected with the indicated siRNAs and treated with 100 pM of TGF-β for 24 h. Cells were treated with 10 μM 5-bromo-2′-deoxyuridine (BrdU) at 37 °C for 1 h. BrdU-incorporated cells were detected by flow cytometry after being fixed with paraformaldehyde, permeabilized with saponin, and stained with anti-BrdU FITC. Results are shown as means ± S.D. (n = 3). Significant differences are indicated as **p < 0.01.

Since our results suggested the involvement of SET8 in the repression of TGF-β signaling, we investigated whether the depletion of SET8 affected the biological effects of TGF-β. TGF-β has been shown to exert cytostatic effects on human immortalized keratinocyte HaCaT cells^31^. We knocked down the expression of SET8 by siRNA in HaCaT cells. As shown in Fig. 4A and B, the depletion of SET8 increased TGF-β-induced PAI-1 and p21 expression, which are effectors for TGF-β-mediated cytostatic responses^32^. In addition, knockdown by shRNA targeting different sequences of SET8 enhanced the induction of *PAI-1* mRNA by TGF-β, but there was a trend toward induction of *p21* mRNA, but not significantly (Fig. S3). We next investigated whether the knockdown of SET8 affected the antiproliferative effects of TGF-β. The TGF-β treatment inhibited cell proliferation, and its antiproliferative effects were augmented by the depletion of SET8 (Fig. 4C). Moreover, the results of the BrdU incorporation assay showed that the depletion of SET8 enhanced TGF-β-induced G1-phase arrest (Fig. 4D). Collectively, these results demonstrated that SET8 negatively regulated the TGF-β-induced expression of PAI-1 and p21 and inhibited the antiproliferative effects of TGF-β.

### SET8 occupies PAI-1 and p21 promoter regions in a competitive manner against p300

**Figure 5:**
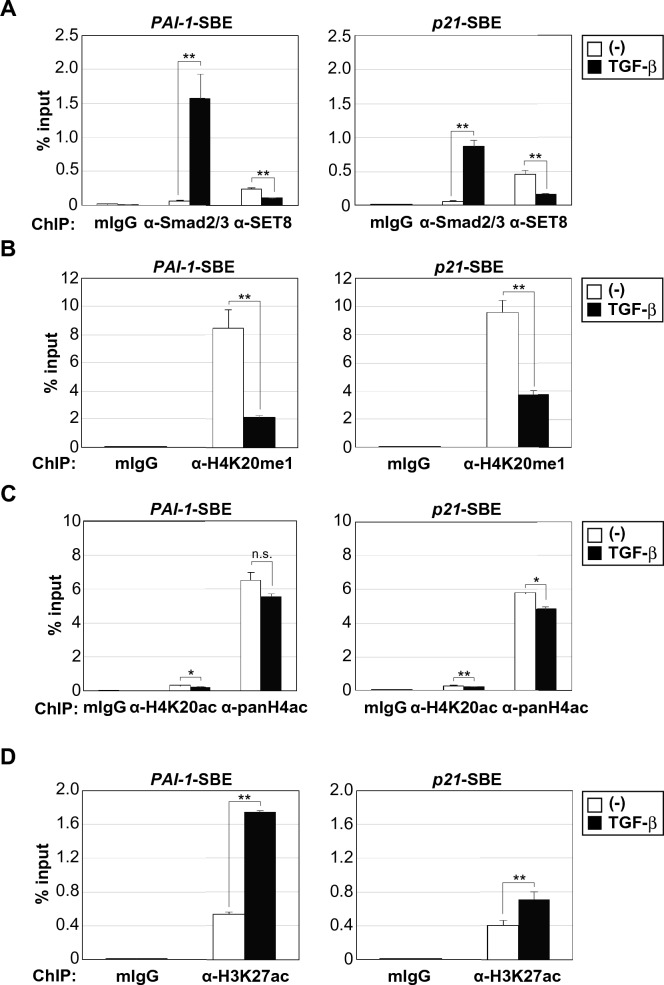
SET8 resides on the *PAI-1* and *p21* promoters before TGF-β stimulation and promoter-bound SET8 decreases in response to TGF-β. (**A**–**D**) HaCaT cells were treated with 40 pM of TGF-β for 1.5 h. Cell lysates were subjected to ChIP assays with the indicated antibodies. Extracted DNA fragments were analyzed by real-time PCR. Results are shown as means ± S.D. (n = 3). Significant differences are indicated as **p < 0.01, *p < 0.05, n.s.: not significant.

To elucidate the mechanisms underlying SET8-mediated transcriptional regulation, we performed ChIP assays to examine the recruitment of SET8 to TGF-β target promoters. We treated HaCaT cells with TGF-β for 1.5 h and examined the binding of Smad2/3 and SET8 to the *PAI-1* and *p21* promoters. As expected, Smad2/3 was recruited to these promoters after the TGF-β treatment (Fig. 5A). SET8 resided on the promoters even before the stimulation and promoter-bound SET8 decreased in response to the TGF-β treatment (Fig. 5A). With the withdrawal of SET8 from these promoters, the levels of H4K20me1, a downstream target of SET8, were also significantly decreased by the TGF-β treatment (Fig. 5B). Histone tail modifications have been shown to play a fundamental role in chromatin compaction, nucleosome dynamics, and transcription^33^. Since numerous studies have demonstrated the interplay between histone methylation and acetylation in transcriptional regulation^34^, we examined histone H4 acetylation levels at the *PAI-1* and *p21* promoters using the anti-acetylated histone H4K20 (H4K20ac) antibody and anti-histone H4 pan-acetyl (panH4ac) antibody. However, the TGF-β treatment only negligibly affected H4K20ac levels and global H4 acetylation levels (Fig. 5C). These results indicate that a decrease in H4K20me1 on these promoters did not increase H4K20ac or the overall acetylation of histone H4.

**Figure 6:**
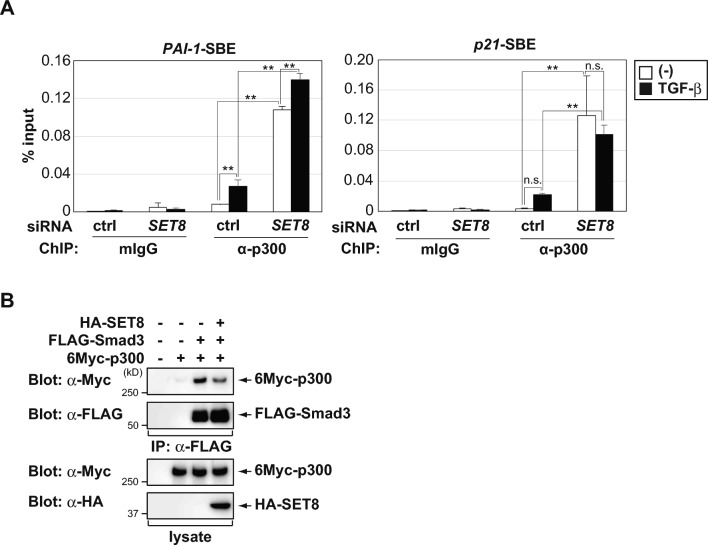
SET8 occupies *PAI-1* and *p21* promoter regions in a competitive manner against p300. (**A**) HaCaT cells were transiently transfected with the indicated siRNAs. After 48 h, cells were treated with 40 pM of TGF-β for 1.5 h. Cell lysates were subjected to ChIP assays with the indicated antibodies. Extracted DNA fragments were analyzed by real-time PCR. Results are shown as means ± S.D. (n = 3). (**B**) HEK293 cells were transfected with the indicated constructs. After 24 h, FLAG-Smad3 was immunoprecipitated (IP) with an anti-FLAG antibody and then immunoblotted with the indicated antibodies. Significant differences are indicated as **p < 0.01, n.s.: not significant.

**Figure 7:**
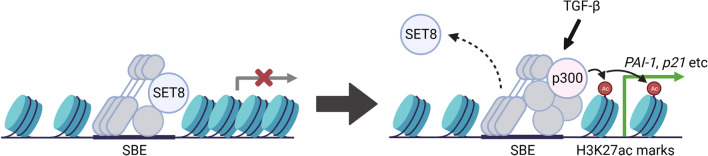
A schematic representation for the SET8-mediated suppression of TGF-β-induced transcriptional activation. In the steady state, SET8 localizes to the Smad-binding region on chromatin and represses transcription. In the presence of TGF-β, SET8 dissociates from the promoter, causing transcriptional activation with H3K27 acetylation by the Smad-p300 complex. This illustration was created by BioRender software (https://www.biorender.com).

Histone H3 and H4 are major targets for acetylation, and specific combinations of acetyl and methyl modifications in these histone tails may act synergistically to regulate transcription^35^. Therefore, we focused on histone H3 acetylation levels at the *PAI-1* and *p21* promoters and found that the TGF-β treatment significantly increased acetylated histone H3K27 (H3K27ac) levels (Fig. 5D). The histone acetyltransferase p300 is a general transcriptional co-activator that introduces the H3K27ac mark on enhancers, thereby triggering their activation and gene transcription, and it has been shown to interact with Smad2/3 in order to promote Smad-dependent transcription^36–39^. As shown in Fig. 6A, TGF-β treatment increased the level of p300 binding to the *PAI-1* promoter, while there was a trend but no significant for p300 binding to the *p21* promoter. We also found that the knockdown of SET8 promoted the recruitment of p300 to the *PAI-1* promoter region in the absence and presence of TGF-β. The recruitment of p300 to the *p21* promoter was increased by the knockdown of SET8, but was not further increased by TGF-β. Furthermore, SET8 expression attenuated the interaction between Smad3 and p300 (Fig. 6B). Based on these results, one mechanism for SET8-mediated transcriptional regulation may be SET8 binding to the *PAI-1* and *p21* promoters and the TGF-β treatment reducing SET8 levels on these promoters in an attempt to promote gene transcription. SET8 may occupy promoter regions in a competitive manner against p300, and p300 attenuates the suppressive binding of SET8 on these promoters to facilitate gene transcription through H3K27ac (Fig. 7).

## Discussion

TGF-β regulates a diverse set of cellular processes, including cell proliferation, migration, differentiation, and apoptosis. TGF-β signaling is tightly regulated at different levels along its pathway, and its deregulation has been associated with various diseases, such as cancer. Previous studies described negative regulators of TGF-β signaling as major drivers of tumor progression. Ikushima et al*.* identified human homologue of Maid (HHM) as a negative regulator of TGF-β signaling^40^. HHM disrupts the physical interaction of Smad2/3 with Olig1, a Smad-binding transcription factor, to suppress TGF-β-induced growth inhibition. Wang et al*.* reported that EWI-2 suppressed TGF-β signaling by decreasing phosphorylated-Smad2 levels, leading to changes in melanoma growth and metastasis^41^. In the present study, we identified SET8 as a negative regulator of TGF-β signaling. We also demonstrated that the depletion of SET8 augmented the antiproliferative effects of TGF-β (Fig. 4C and D). These results contribute to our knowledge of the mechanisms underlying aberrant TGF-β signaling in cancer.

The pleiotropic outcome of the TGF-β signaling pathway relies on the control of TGF-β target gene expression, which depends on the functions of Smads proteins along with those of diverse modulators, such as transcriptional factors and cofactors^15^. We herein clearly demonstrated that the TGF-β treatment enhanced the recruitment of p300 to the *PAI-1* and *p21* promoters, while the depletion of SET8 augmented its recruitment to these promoter regions (Fig. 6A). However, other transcriptional coactivators may participate in the physical interplay between Smad2/3 and SET8, thereby facilitating TGF-β-induced *PAI-1* and *p21* transcription. Previous studies reported that histone acetyltransferase CREB-binding protein (CBP) associated with Smad3 and stimulated TGF-β-induced transcription^37,38^. Similarly, p300/CBP-associated factor, a histone acetyltransferase, was shown to directly bind to Smad3 and activate Smad-mediated transcriptional responses^42^. Therefore, TGF-β may also induce the replacement of the suppressive binding of SET8 with other coactivators to promote gene transcription.

SET8 is involved in vital cellular processes, such as DNA replication, mitosis, and DNA damage repair, indicating its importance in cell proliferation^21^. Accumulating evidence has suggested a relationship between SET8 and tumorigenesis. For example, SET8 was shown to negatively regulate p53 activity through its monomethylation at lysine 382 and the knockdown of SET8 promoted p53-mediated cell cycle arrest and apoptosis^18^. SET8 promoted the transcription of Wnt target genes via its H4K20 monomethylation activity^43^. Moreover, SET8 was found to be overexpressed in various cancers, including bladder cancer, non-small cell and small cell lung carcinoma, hepatocellular carcinoma, and pancreatic cancer^20^. Since cancer cells evade the tumor-suppressive effects of TGF-β and exploit it to maintain motility or malignancy, many human cancers reportedly have genetically inactive mutations in TGF-β receptors or Smads. Therefore, the temporal repression of TGF-β signaling through the overexpression of SET8 may provide tumor cells with an advantage. In the present study, we showed that SET8 down-regulated the expression of PAI-1 and p21 and evaded the antiproliferative effects of TGF-β. Based on the present results, SET8 largely functions as an oncogene and has potential as a target in cancer therapy.

Histone tail modifications play an essential role in chromatin compaction, nucleosome dynamics, and transcription^33^. Recent studies reported the involvement of histone methylation in transcription regulation^44^. We herein revealed that the TGF-β treatment significantly decreased H4K20me1 levels with the withdrawal of SET8 from the *PAI-1* and *p21* promoters (Fig. 5A); however, SET8 methyltransferase activity appeared to be dispensable for the repression of TGF-β target genes (Fig. 3D). The SET8-mediated H4K20me1 mark is reportedly involved in both the activation and repression of transcription^21^. On the other hand, some studies indicated that SET8-mediated H4K20 methylation was unlikely to play a major role in transcription regulation because the H4K20me1 mark did not significantly affect the GAL4 reporter or NF-κB pathway^17,43^. Different reader proteins of H4K20me1 may contribute to the diverse biological effects of H4K20me1. The reading of the H4K20me1 mark by different effectors, in turn, may be affected by site-specific DNA and other transcription cofactors.

The deletion of SET8 was found to inhibit PAI-1 and p21 mRNA and protein expression, suppress cell growth, and promote the binding of p300 to *PAI-1* and *p21* promoters without TGF-β stimulation, suggesting the existence of a TGF-β-independent mechanism of transcriptional regulation of these genes by SET8. Since it has been reported that p53 induces *p21* and *PAI-1* transcription^45^, while p53 function is suppressed by SET8^18^, the induction of PAI-1 and p21 by SET8 knockdown could be partially explained. On the other hand, HaCaT cells have two mutant *TP53* alleles, which impairs p53 function, but p63 and p73, members of the p53 family, may be regulated by SET8 in the same way as p53. We have previously reported that p63 knockdown resulted in suppression of TGF-β-induced PAI-1 expression in HaCaT cells^46^. KLF4 is a transcription factor that induces *p21* transcription^47^, and SET8 binds to and inactivates KLF4^48^, suggesting the induction of p21 by SET8 knockdown might be mediated by KLF4. Further studies are necessary to clarify these concerns.

Collectively, the present results revealed that SET8 negatively affected TGF-β target promoters in a catalytic activity-independent manner. SET8 down-regulated the TGF-β-induced expression of PAI-1 and p21 and repressed the antiproliferative effects of TGF-β. Mechanistically, SET8 occupied the *PAI-1* and *p21* promoters, and the TGF-β treatment triggered the replacement of the suppressive binding of SET8 with p300 on these promoters, possibly in an attempt to promote gene transcription. Since resistance to the antiproliferative effects of TGF-β is linked to malignant transformation, SET8 has potential as a target for therapeutic interventions.

## Materials and methods

### Cell lines, plasmids, and transfection

COS7, HEK293, HepG2, and HaCaT cells were cultured in high-glucose Dulbecco’s modified Eagle’s medium (Sigma, St. Louis, MO, USA) supplemented with 10% heat-inactivated fetal bovine serum (FBS) (Sigma), 100 U/ml of penicillin G, and 100 µg/ml of streptomycin at 37 °C in the presence of 5% CO_2_. H1299 cells were cultured in Roswell Park Memorial Institute 1640 medium (Sigma) containing 10% FBS and penicillin/streptomycin.

The original constructs encoding Smad3, ALK5 (T204D), SET8, and p300 were previously described^49,50^. Human SETDs and SMYDs were cloned from the complementary DNA (cDNA) of MCF7 cells into pcDNA3/FLAG. Regarding short-hairpin RNA (shRNA)-mediated gene silencing, gene-specific hairpin oligonucleotides under the H1 promoter were ligated into the pLenti6/V5-DEST vector (Invitrogen, Carlsbad, CA, USA)^51^. The sequence for *SET8* shRNA was 5′-GGAAGAGAACTCAGTTACA-3′^29^.

Plasmids for DNA transfection were transiently transfected with Lipofectamine 2000 (Invitrogen). siRNAs for short interfering RNA (siRNA) transfection were transfected using Lipofectamine RNAiMAX reagent (Invitrogen) according to the manufacturer’s protocol. Human *SET8* siRNA (sense, 5′-CAAAUGCUCUGGAAUGCGUdTdT-3′)^29^ was purchased from Sigma. Stealth RNAi™ siRNA Negative Control Med GC Duplex was obtained from Invitrogen.

### Immunochemical methods and antibodies

Protein solubilization, immunoblotting, and immunoprecipitation were conducted as previously reported^52^. The following commercially available antibodies were used: anti-PAI-1 (clone 41/PAI-1; BD Biosciences, Franklin Lakes, NJ, USA), anti-β-actin (AC-15; Sigma), anti-Smad2/3 (clone 18/Smad2/3; BD Bioscience), anti-Myc (4A6; Sigma), anti-FLAG (M2; Sigma), anti-SET8 (06-1304; Sigma), anti-HA (Y-11; Santa Cruz Biotechnology, Dallas, TX, USA), anti-GST (B-14, Santa Cruz Biotechnology), anti-SET8 (D-11, Santa Cruz Biotechnology), anti-SET8 (C18B7, Cell Signaling Technology, Beverly, MA), anti-Smad2 (D43B4, Cell Signaling Technology), anti-phospho-Smad2 (Ser465/467) (138D4, Cell Signaling Technology), anti-Smad3 (C67H9, Cell Signaling Technology), anti-phospho-Smad3 (Ser423/425) (C25A9, Cell Signaling Technology), anti-Smad2/3 (D7G7, Cell Signaling Technology), anti-p21 (clone70/Cip1/WAF1; BD Bioscience), anti-histone H4K20me1 (MABI0421, MBL, Nagoya, Japan), anti-histone H4K20ac (MABI0420, MBL), anti-pan histone H4ac (MABI0430, MBL), anti-histone H3K27ac (MABI0309, MBL), and anti-p300 (F-4; Santa Cruz Biotechnology). Mouse immunoglobulin G1 (IgG1) (MB002; R & D Systems, Minneapolis, MN, USA) was used as a control. The following secondary antibodies purchased from Jackson ImmunoResearch Inc. (West Grove, PA, USA) were used: anti-mouse IgG-horseradish peroxidase (HRP) (115-035-003), anti-rabbit IgG-HRP (111-035-003), and anti-rabbit-IgG (light chain specific)-HRP (211-032-171; for immunoblotting the immunoprecipitates).

### GST pull-down assay

GST fusion proteins were purified as previously reported^50^. cDNA encoding Smad3 was cloned into pCold I (TaKaRa Bio Inc., Shiga, Japan) and expressed in the *Escherichia coli* BL21 (DE3) strain, and His-tagged Smad3 was purified as recommended in the instructions (Qiagen, Hilden, Germany). Cells were subjected to lysis in TNTE buffer (50 mM Tris–HCl (pH 7.5), 150 mM NaCl, 1 mM EDTA, and 0.5% Triton X-100) supplemented with protease inhibitors and pulled down with GST fusion proteins. Samples were then subjected to immunoblotting.

### Luciferase assay

Cells were transfected with the luciferase reporter plasmid, expression plasmids, β-gal expression plasmid, and empty vector. The total amount of transfected DNA was the same in each experiment. Luciferase activity in cell lysates was measured and normalized against β-gal activity^46^.

### RNA extraction, reverse transcription, and quantitative PCR (qPCR)

Total RNA was extracted as previously described^52^. cDNA was then synthesized from total RNA using the ReverTra qPCR RT Master Mix (TOYOBO, Osaka, Japan) according to the manufacturer’s instructions. qPCR was performed using TB Green Premix Ex Taq II (TaKaRa Bio Inc.) and the ABI Prism 7300 sequence detection system (Applied Biosystems, South San Francisco, CA, USA). The specificities of the detected signals were confirmed by a dissociation curve consisting of a single peak. HPRT1 was used as the internal control. The following primer sequences were used: human *PAI-1*, 5′-GGCTGACTTCACGAGTCTTTCA-3′ (forward) and 5′-ATGCGGGCTGAGACTATGACA-3′ (reverse); human *SET8,* 5′-AAGGTGGACTTGAACAGATG-3′ (forward) and 5′-ACCTGTGCTGAGTCTTTGAC-3′ (reverse); human *HPRT1*, 5′-TTTGCTTTCCTTGGTCAGGC-3′ (forward) and 5′-GCTTGCGACCTTGACCATCT-3′ (reverse); human *p21*, 5′-GATTTCTACCACTCCAAACGCC-3′ (forward) and 5′-AGAAGATGTAGAGCGGGC-3′ (reverse).

### Cell viability assay and BrdU incorporation assay

Cell viability was measured using Cell Counting Kit-8 according to the manufacturer’s instructions (Dojindo, Kumamoto, Japan). Cells were transiently transfected with the indicated siRNAs. After transfection, cells were trypsinized and replated at a concentration of 2 × 10^3^ cells per well in a 96-well plate. After 72 h, the WST-8 reagent was added, and cells were incubated at 37 °C for 3 h in a humidified atmosphere of 5% CO_2_. Absorbance at 450 nm of the medium was assessed^52^.

To measure the levels of DNA synthesized, cells were treated with 10 µM 5-bromo-2′-deoxyuridine (BrdU) at 37 °C for 1 h. BrdU-incorporated cells (the S phase) were detected by flow cytometry (FACSVerse, BD Biosciences) after being fixed with paraformaldehyde, permeabilized with saponin, and stained with anti-BrdU FITC (FITC BrdU Flow kit, BD Biosciences) according to the manufacturer’s protocol^52^.

### Chromatin immunoprecipitation (ChIP) assay.

A ChIP assay was performed as previously reported^53,54^. Purified DNA was analyzed by quantitative real-time PCR. Quantitative real-time PCR was performed using GeneAce SYBR qPCR Mix α (Nippon Gene, Tokyo, Japan) and a 7300 Real-Time PCR System (Applied Biosystems). Regarding amplification in real-time PCR, the following primer sequences were used: human *PAI-1* promoter (SBE), 5′-GCAGGACATCCGGGAGAGA-3′ (forward) and 5′-CCAATAGCCTTGGCCTGAGA-3′ (reverse); human *p21* promoter (SBE), 5′-GAGGAAAAGCATCTTGGAG-3′ (forward) and 5′-AAATAGACGGGAGCAACG-3′ (reverse); and human *HPRT1* first intron, 5′-TGTTTGGGCTATTTACTAGTTG-3′ (forward) and 5′-ATAAAATGACTTAAGCCCAGAG-3′ (reverse).

### Statistical analysis

The significance of differences between two groups was evaluated using a two-tailed Student’s *t*-test. In multigroup analyses, significance was assessed using a one-way analysis of variance with the post-hoc Tukey–Kramer honestly significant difference test.

### Supplementary Information

Supplementary Figures.

## Data Availability

The datasets generated and analyzed during the current study are available from the corresponding author on reasonable request.
