# Supplementary material for: SET8 is a novel negative regulator of TGF-β signaling in a methylation-independent manner
Source: Sci Rep. 2023 Dec 18;13:22877. doi: 10.1038/s41598-023-49961-x (PMC10739863; doi:10.1038/s41598-023-49961-x)
Supplement: Supplementary file 1 — Supplementary Figures. [file 41598_2023_49961_MOESM1_ESM.pdf]

SET8 is a novel negative regulator of TGF-β signaling in a methylation-independent manner

Mai Nagasaka[1,#], Yasumichi Inoue[1,#,*], Yuji Nagao[1], Chiharu Miyajima[1], Daisuke Morishita[1], Hiromasa Aoki[2], Mineyoshi Aoyama[2], Takeshi Imamura[3], Hidetoshi Hayashi[1,*]

[1]Department of Cell Signaling, Graduate School of Pharmaceutical Sciences, Nagoya City University; 467-8603 Nagoya, Japan.

[2]Department of Pathobiology, Graduate School of Pharmaceutical Sciences, Nagoya City University; Nagoya 467-8603, Japan.

[3]Department of Molecular Medicine for Pathogenesis, Graduate School of Medicine, Ehime University; Ehime 791-0295, Japan

[#]These authors contributed equally to this work.

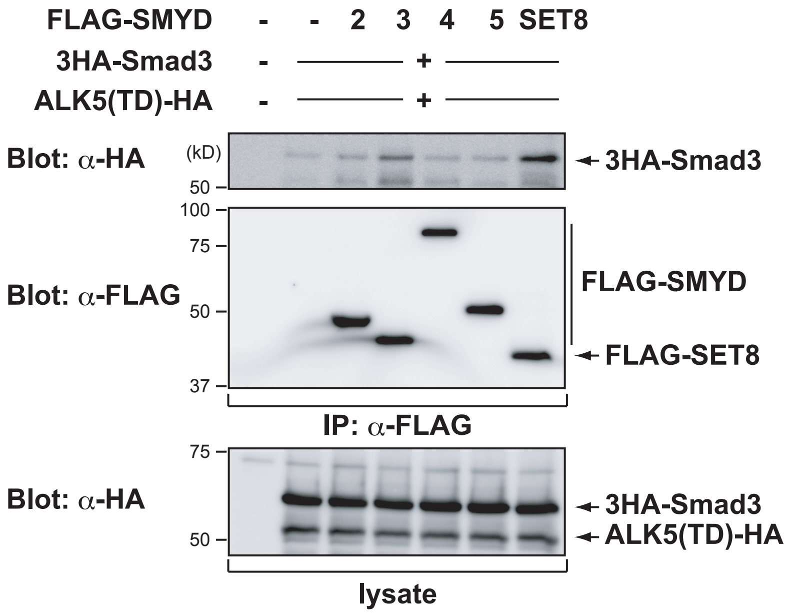

Supplementary Figure S1. SMYD family proteins do not associate with Smad3.
COS7 cells were transiently transfected with the indicated constructs. After 24 h, cell lysates were immunoprecipitated (IP) with an anti-FLAG antibody and then immunoblotted with the indicated antibodies.

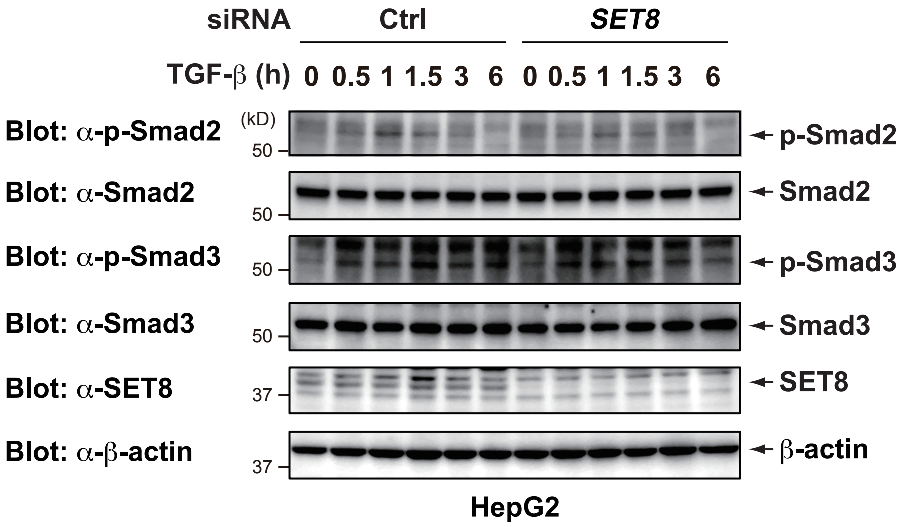

Supplementary Figure S2. TGF-β induced similar levels of Smad2 and Smad3 phosphorylation in both control and SET8 knockdown cells.
HepG2 cells were transiently transfected with the indicated siRNAs. After 48 h, cells were treated with 100 pM of TGF-β for the indicated periods. The cell lysates were immunoblotted with the indicated antibodies.

Supplementary Figure S2

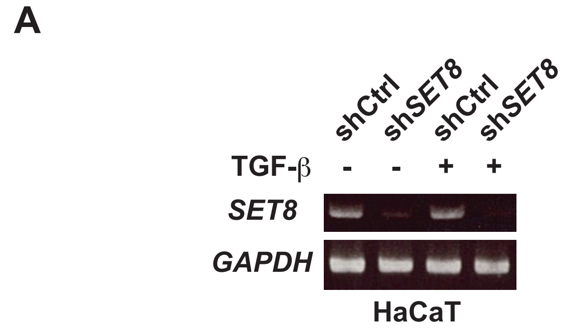

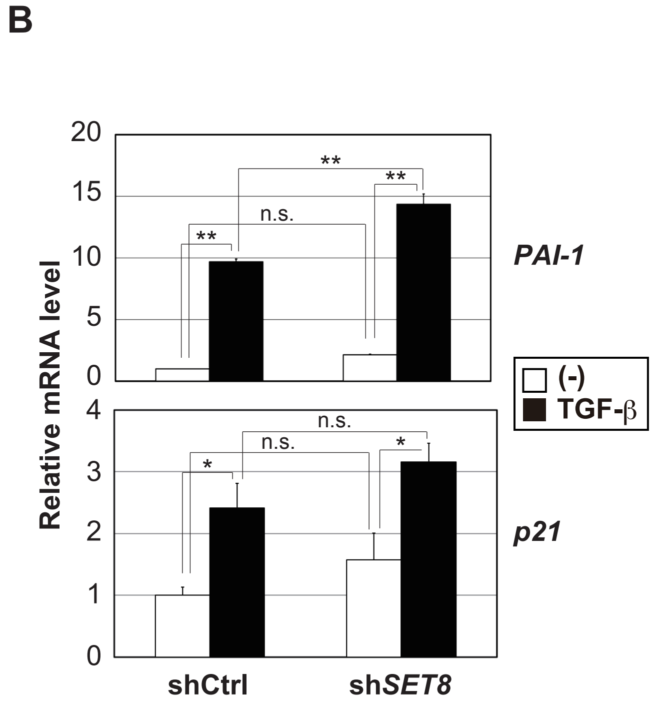

Supplementary Figure S3. SET8 down-regulates the TGF-β-induced expression of PAI-1 and p21 and represses antiproliferative effects of TGF-β.

(A) HaCaT cells were transfected with the indicated shRNAs. After 96 h, cells were treated with 40 pM of TGF-β for 6 h. The expression of each gene was assessed by semi-quantitative PCR.

(B) HaCaT cells were transfected with the indicated shRNAs. After 96 h, cells were treated with 40 pM of TGF-β for 6 h. The expression of each gene was assessed by quantitative PCR, and the mRNA levels of the indicated genes were normalized with *HPRT1* mRNA. Results are shown as means ± S.D. (n=2).

Significant differences are indicated as $^{**}p < 0.01$, $^{*}p < 0.05$, n.s.: not significant.

Supplementary Figure S3

Fig. 1A

IP: α-FLAG Blot: α-HA

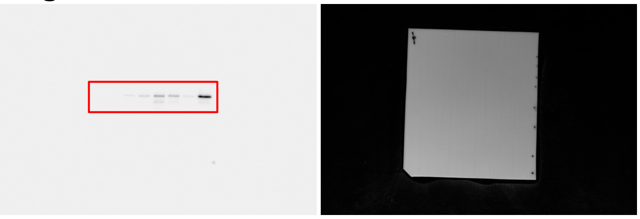

IP: α-FLAG Blot: α-FLAG

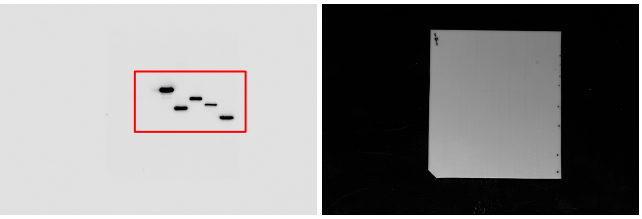

lysate Blot: α-HA

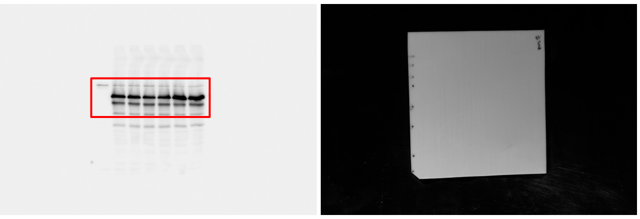

Fig. 1B

IP: α-FLAG Blot: α-SET8

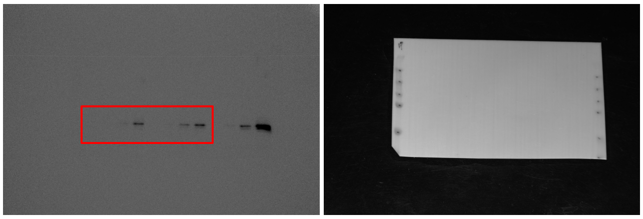

IP: α-FLAG Blot: α-FLAG

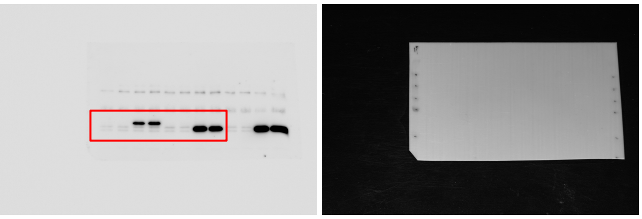

lysate Blot: α-Myc

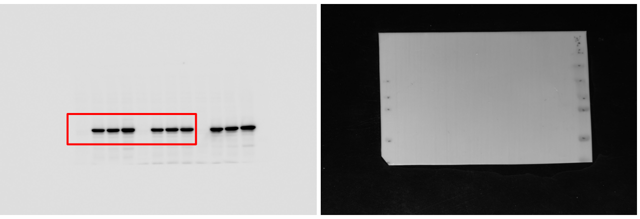

lysate Blot: α-HA

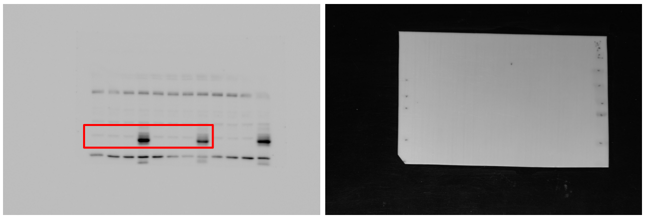

Fig. 1C

IP: α-Smad2/3 Blot: α-SET8

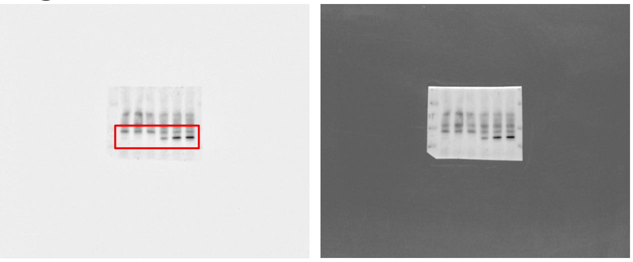

IP: α-Smad2/3 Blot: α-Smad2/3

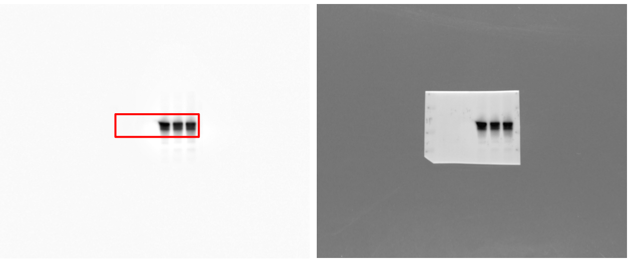

lysate Blot: α-SET8

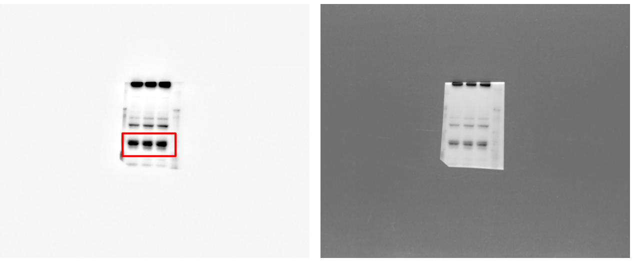

lysate Blot: α-β-actin

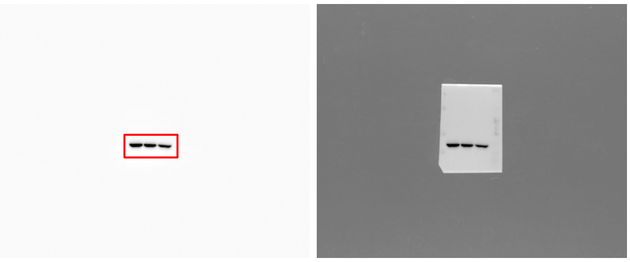

Supplementary Figure S4. Uncropped images of gels/blots.
Red boxes indicate the parts that are shown in the figures. Please note that some membranes were cut into multiple strips prior to immunoblotting to detect multiple antigens.

Fig. 1D

IP: α-FLAG Blot: α-SET8

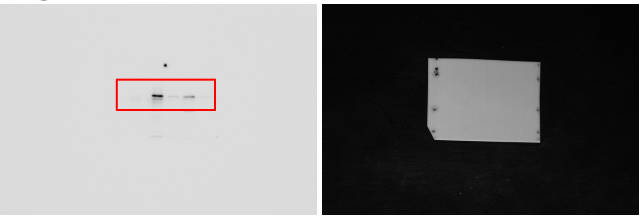

IP: α-FLAG Blot: α-FLAG

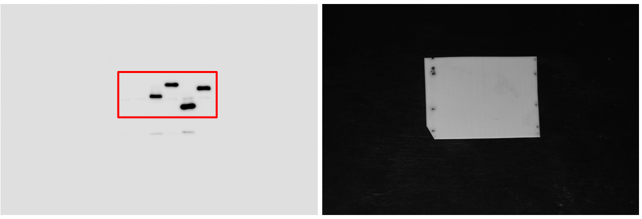

lysate Blot: α-Myc

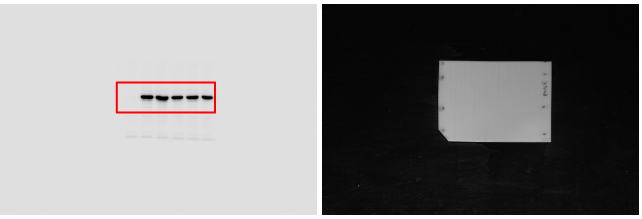

Fig. 2B

Blot: α-Smad2/3

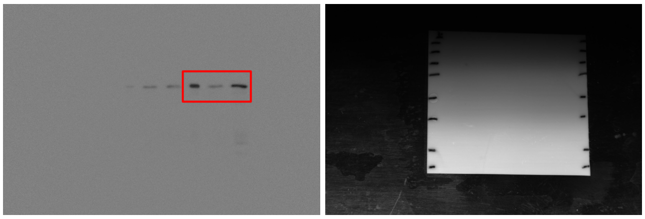

Blot: α-GST

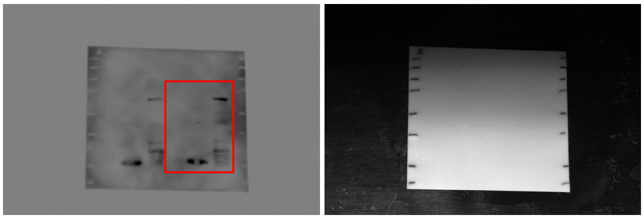

Fig. 2C

Blot: α-FLAG

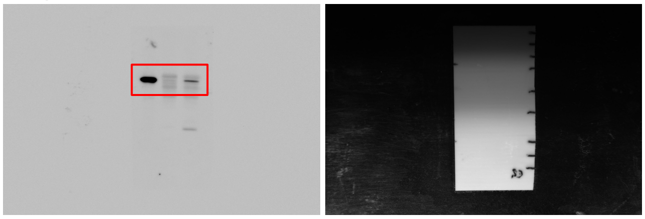

Blot: α-GST

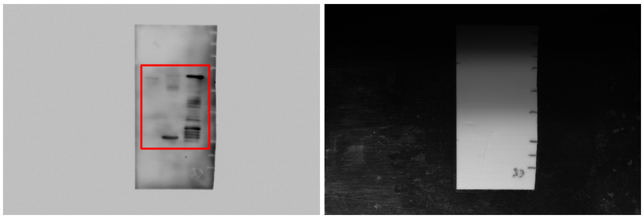

Blot: α-FLAG

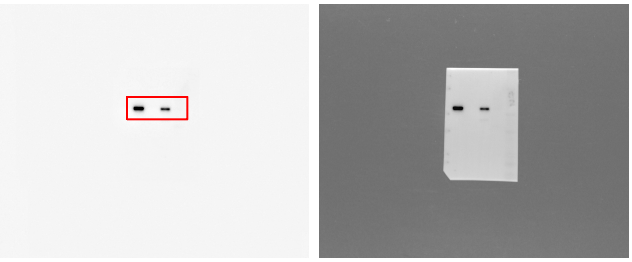

Blot: α-GST

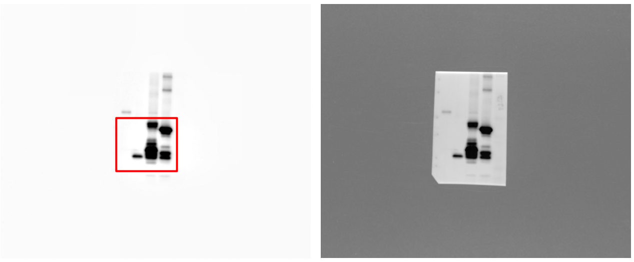

Supplementary Figure S4 continued

Fig. 2D

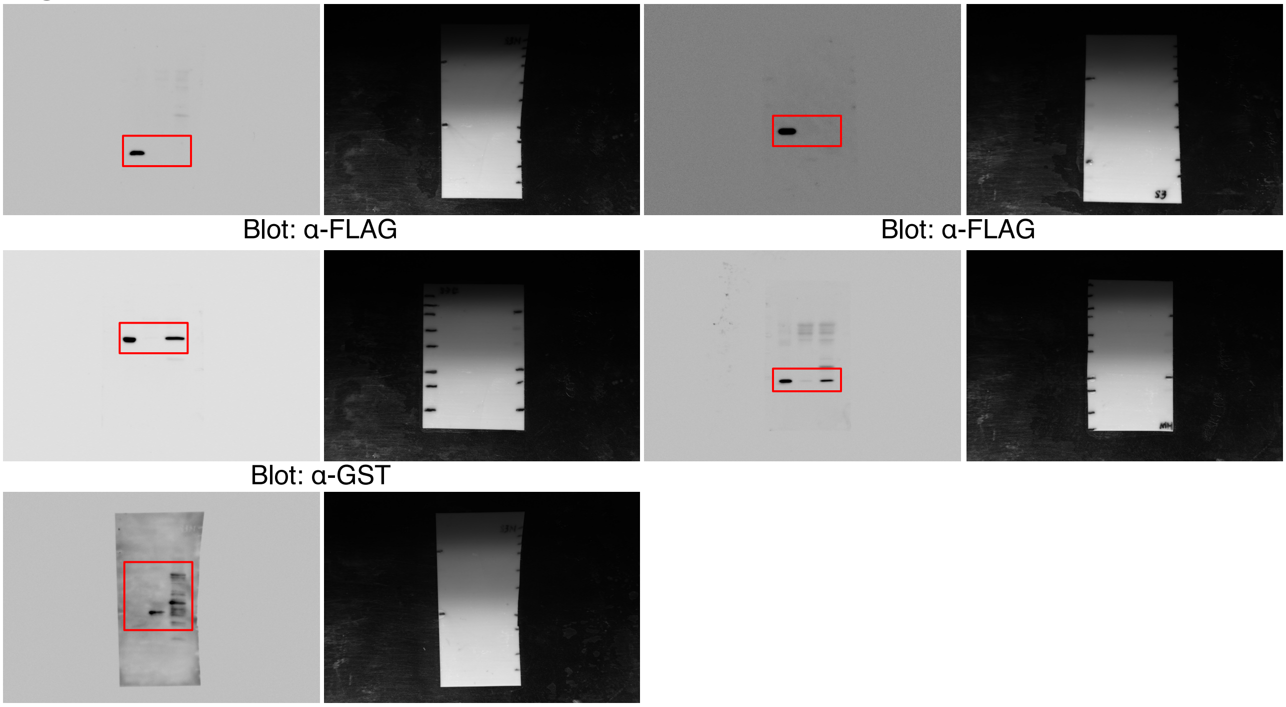

Fig. 3A

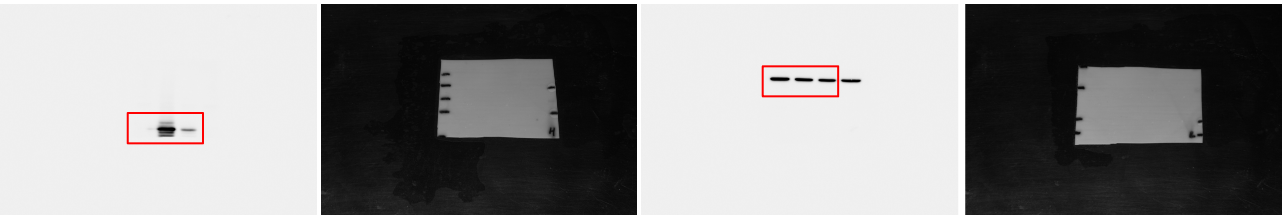

Fig. 4A

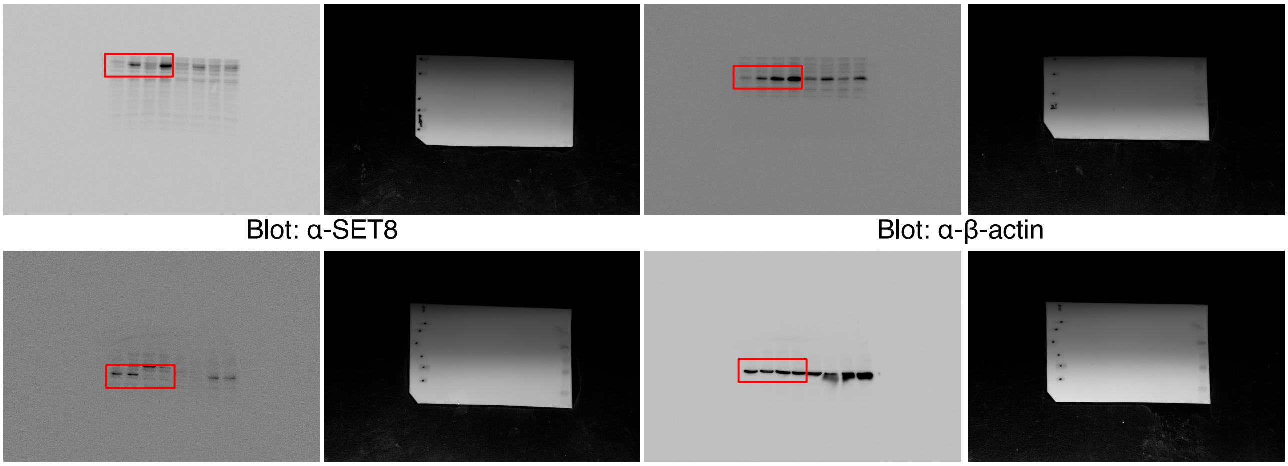

Supplementary Figure S4 continued

Fig. 6B

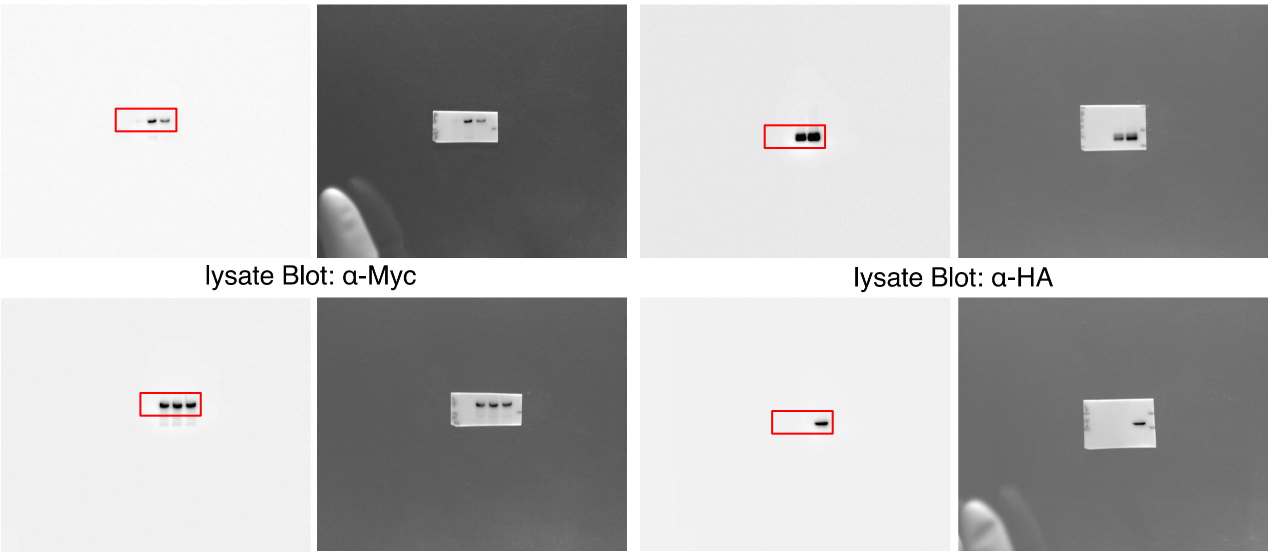

Fig. S1

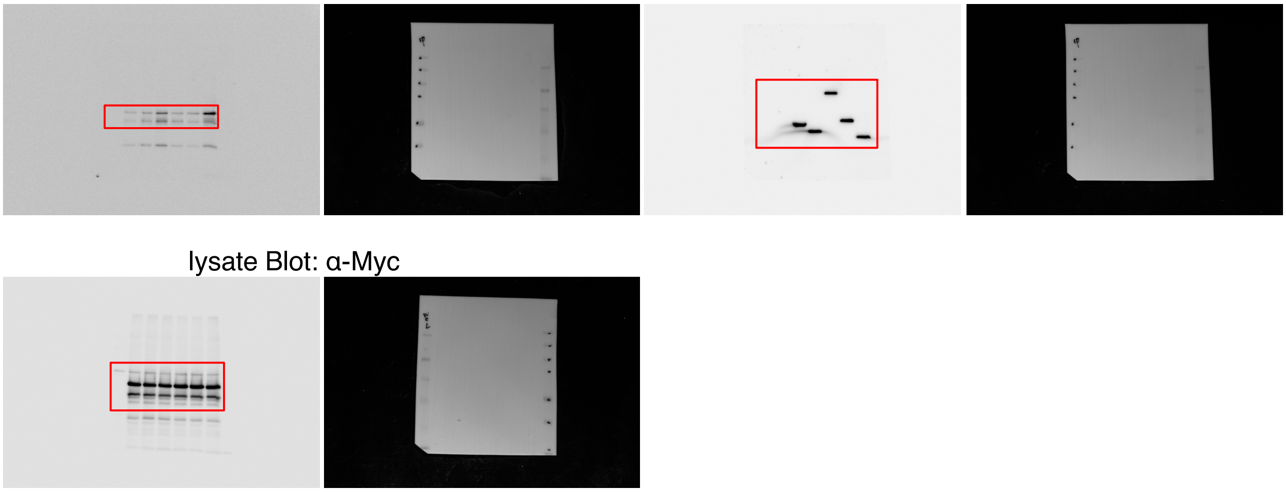

Fig. S3A

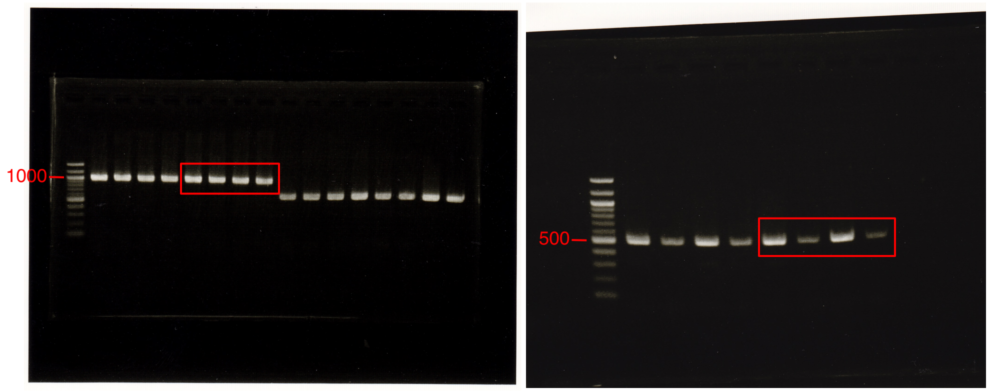

Supplementary Figure S4 continued

Fig. S2

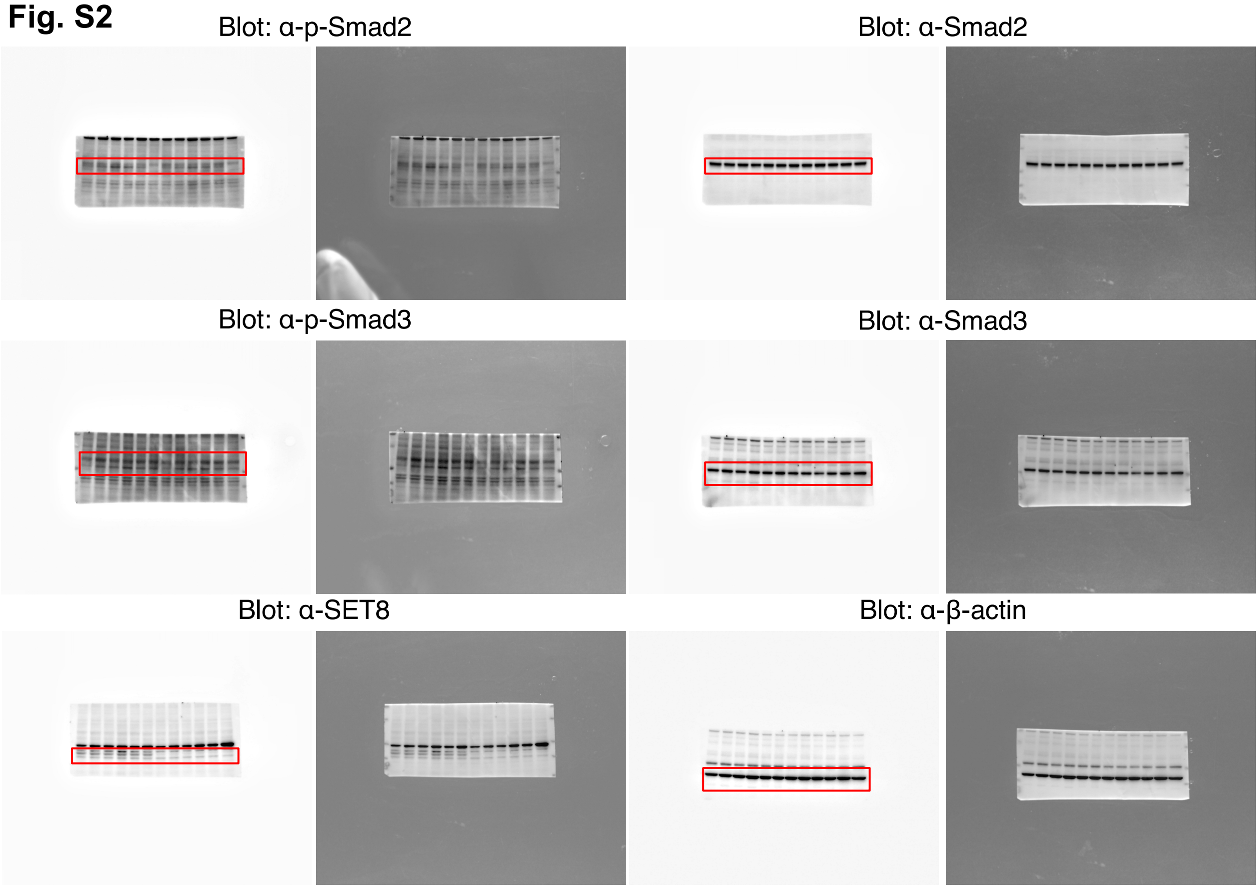

Supplementary Figure S4 continued
